# Research Progress on the NSP9 Protein of Porcine Reproductive and Respiratory Syndrome Virus

**DOI:** 10.3389/fvets.2022.872205

**Published:** 2022-07-11

**Authors:** Huiyang Sha, Hang Zhang, Yao Chen, Liangzong Huang, Mengmeng Zhao, Nina Wang

**Affiliations:** ^1^Department of Veterinary Medicine, School of Life Science and Engineering, Foshan University, Foshan, China; ^2^Veterinary Teaching Hospital, Foshan University, Foshan, China

**Keywords:** PRRSV, NSP9, RdRp, protein interaction, pathogenicity

## Abstract

Porcine reproductive and respiratory syndrome (PRRS) is a contagious disease caused by the porcine reproductive and respiratory syndrome virus (PRRSV). PRRS is also called “blue ear disease” because of the characteristic blue ear in infected sows and piglets. Its main clinical features are reproductive disorders of sows, breathing difficulties in piglets, and fattening in pigs, which cause considerable losses to the swine industry. NSP9, a non-structural protein of PRRSV, plays a vital role in PRRSV replication and virulence because of its RNA-dependent RNA polymerase (RdRp) structure. The NSP9 sequence is highly conserved and contains T cell epitopes, which are beneficial for the development of future vaccines. NSP9 acts as the protein interaction hub between virus and host during PRRSV infection, especially in RNA replication and transcription. Herein, we comprehensively review the application of NSP9 in terms of genetic evolution analysis, interaction with host proteins that affect virus replication, interaction with other viral proteins, pathogenicity, regulation of cellular immune response, antiviral drugs, vaccines, and detection methods. This review can therefore provide innovative ideas and strategies for PRRSV prevention and control.

## Introduction

Porcine reproductive and respiratory syndrome (PRRS) is a highly contagious and virulent infectious disease caused by the porcine reproductive and respiratory syndrome virus (PRRSV). PRRS is mainly characterized by reproductive disorders in sows and breathing difficulties in pigs of all ages. It leads to substantial losses in the global swine industry. PRRSV belongs to the order Nidovirale, family Arteriaviridae, and is a single-stranded positive-sense RNA virus with an envelope and a non-segmented genome (1). PRRSV escapes the host immunosuppression reaction after infection and inhibits co-infection with other pathogens. PRRSV is known to frequently mutate and recombine, thereby making it difficult to eliminate and control (2).

**Figure 1:**
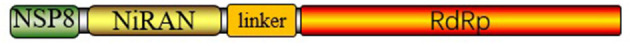
Structure diagram of NSP9 of PRRSV. N-terminal is linked to NSP8 and contains a newly discovered nucleotide transferase domain (NiRAN) related to pancreatic virus RdRp, and C-terminal contains the RdRP domain.

The non-structural protein 9 (NSP9), encoded by ORF1b of PRRSV, is an RNA-dependent RNA polymerase (RdRp). RdRp is essential for genome replication and subgenomic (sg mRNA) synthesis and plays a vital role in viral replication (3). PRRSV RNA synthesis includes genomic RNAs (gRNAs) and sgRNAs and requires a negative-strand RNA strand as a template (4). NSP9 alone or with NSP10 cannot activate the synthesis of virus-negative strand RNA (5). The combination of ORF1a and ORF1b is necessary for synthesizing viral negative-strand RNA (6). In PRRSV-infected MARC-145 cells, NSP9 can be identified as a 72–95 kDa band using NSP9 monoclonal antibodies and NSP8 polyclonal antibodies, respectively, which is higher than the actual molecular weight of NSP9. The migration rate of NSP8-9 expressed *in vitro* is similar to natural NSP9, and NSP9 can co-immunoprecipitate with the NSP8 antibody. NSP4 and NSP2 PLP2 cannot cleave NSP8-9 *in vitro*, which indicates that NSP8 is the N-terminal extension of NSP9 (7). The RdRp domain is located at the C-terminal of the replicase subunit of NSP9, which contains an upstream domain with an unknown function (8). PRRSV NSP9 is thought to contain NSP8 at the N-terminal and RdRp at the C-terminal (9). The N-terminal region also contains a newly discovered nucleotide transferase domain (NiRAN) which is related to the RdRp of the pancreatic virus (Figure 1).

The most common feature of PRRSV NSP9 is its RdRp structure, which is also present in other Arteriviruses (10). All positive-strand RNA viruses encode an RdRp, which acts as a catalytic subunit of virus replication and transcription complexes, and guides the synthesis of viral RNA along with other viral proteins and sometimes host proteins (11). Severe acute respiratory syndrome coronavirus (SARS-CoV) and equine arteritis virus (EAV) also possess an RdRp, which is a ribozyme for the replication and transcription of their multi-protein complexes (12). RdRp is located in an unusually large replicase subunit for positive-strand ribonucleic acid viruses belonging to nested viruses (13). Bioinformatic analysis of this non-structural protein (NSP) revealed a non-viral characteristic domain (genetic marker) located near the N-terminal of RdRp, and no apparent homolog was identified at other sites. This domain exhibits nucleotidase activity based on its conservative spectrum (13). The homologous polymerase subunit (NSP12) of the distantly related coronaviruses has potent primer-dependent RdRp activity (14). Cytoplasmic replication of a positive-strand RNA virus is related to the characteristic virus membrane structure from host organelles. Double-membrane vesicles are related to EAV RNA synthesis, which can be used to immunolabel EAV NSPs (15).

The NSP9 sequence is highly conserved. One main factor in PRRSV virulence is the interaction with the host or viral proteins to regulate viral replication. This interaction is involved in regulating the cellular immune response, and small interfering RNA (siRNA) targeting NSP9 can lead to the inhibition of virus replication. This review focuses on the evolutionary genetic analysis of NSP9, the influence of the interaction between NSP9 and host proteins or other viral proteins on virus replication, the relationship between NSP9 and pathogenicity, regulation of the cellular immune response by NSP9, the influence of compound drugs and modifications of NSP9 on virus replication, and NSP9 vaccine and detection methods to enhance our understanding of PRRSV replication, and promote the research and development of anti-PRRSV drugs and future vaccines.

## Genetic Evolution Analysis of NSP9 Sequence

**Figure 2:**
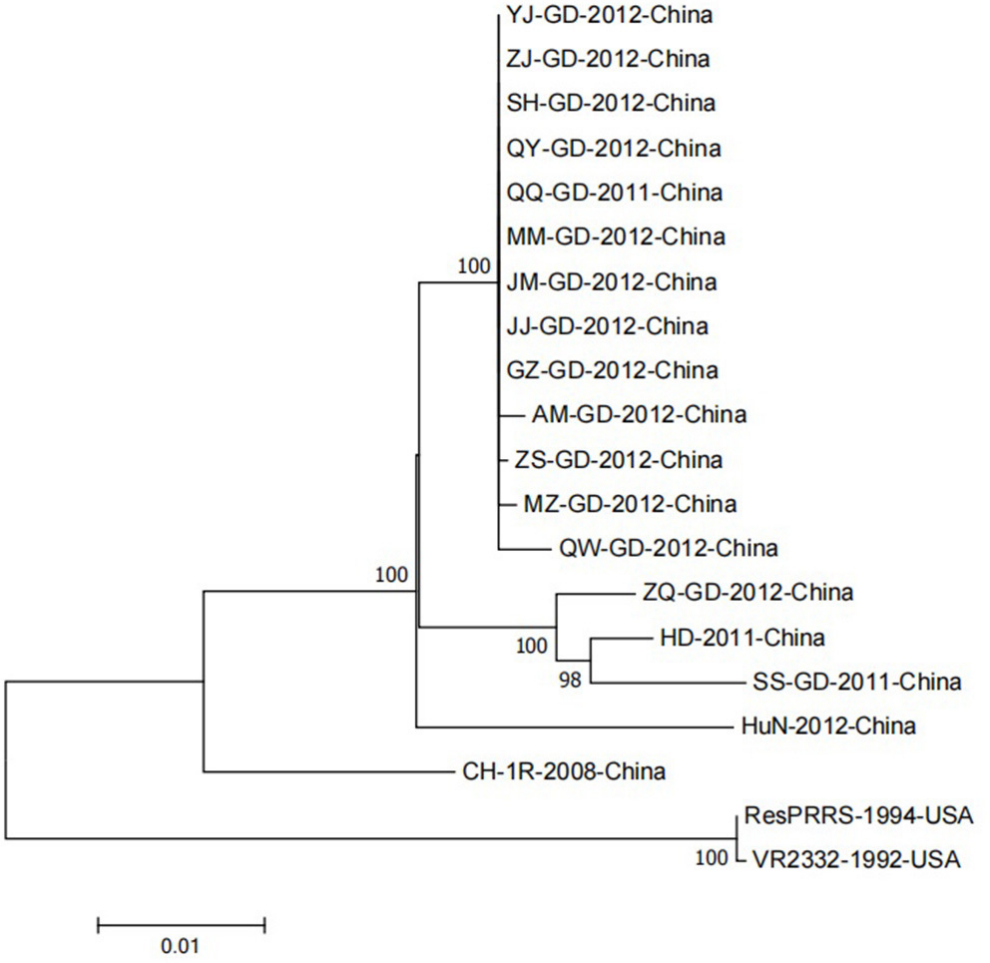
A neighbor-joining phylogenetic tree was constructed based on the NSP9 sequences of 20 PRRSV strains, using MEGA software (ver. 7.0; Center for Evolutionary Medicine and Informatics, Tempe, AZ) with 1000 bootstrap replicates.

Although the NSP9 nucleotide sequence is highly conserved, gene recombination and amino acid changes are observed. Through phylogenetic analysis, Darwich et al. demonstrated that the isolates could be divided into different groups according to the detected ORFs, and found that NSP9 had the highest similarity with the whole genome cluster (16). The NSP9 amino acid homology between the European isolate prototype Lelystad virus (LV) and the North American isolate SDPRRS (01–08) showed a 98.9% similarity, which was the highest among all NSPs (17). Zhao et al. collected 25 PRRSV strains from Guangdong province. Sequence analysis revealed that the homology of the *NSP9* gene was as high as 98.1–100.0% in amino acids 8, 64, 112, 205, and 356, which was higher than that of traditional strains (Ch-1a, BJ-4, VR2332, MLV) (18). Li et al. reported genomic variation distributed in the 5′ untranslated region, NSP1b, NSP2, NSP3, NSP5, NSP7, NSP9, NSP10, GP5, and N regions of PRRSV GDQJ strains isolated in Guangdong Province in China, compared to that in the highly pathogenic PRRSV(HP-PRRSV) strains, which was also isolated in China (19). Many mutations were identified in both virulent and non-virulent strains. Two identical amino acid mutations located at positions 519 and 544 of NSP9 were observed between HP-PRRSV and classical PRRSV strains, respectively. The virulent strain has a serine (S) at position 519 and threonine (T) at position 544. In contrast, the attenuated strain had threonine (T) at position 519 and alanine (A) at 544 (20). Furthermore, the 586th amino acid of NSP9 was a conserved T residue in all HP-PRRSV strains. However, A was identified at this position in low-pathogenicity PRRSV (LP-PRRSV). T is present at position 592 in HB-1/3.9 and its wild-type HB-1(sh)/2002, whereas S is present at this position in all other strains (21). NSP9-containing virus replicase has a conserved characteristic sequence motif, a Ser-Asp-Asp (SDD) sequence, among the RdRps of positive-strand RNA viruses. In an attempt to detect whether the conserved SDD motif can tolerate other conserved motifs of RNA viruses and assess the influence of each residue on the catalytic activity of RdRp, Zhou et al. observed that RdRp could maintain its transcription activity only when the serine in the SDD motif was replaced by glycine (G). The other substitutions were lethal mutations leading to a loss in transcription activity (22). The 20 PRRSV *NSP9* gene reference strains were retrieved from the NCBI nucleotide database as reference sequences for phylogenetic analysis. Detailed information and the GenBank number of PRRSV reference strains are listed in Table 1. To construct the phylogenetic tree, nucleotide sequences of the target gene were aligned using the ClustalX alignment tool using the MEGA software (ver. 7.0; Center for Evolutionary Medicine and Informatics, Tempe, AZ). Phylogenetic trees were constructed using the Neighbor-joining method with 1,000 bootstrap replicates in MEGA7.0 (Figure 2). The 20 PRRSV *NSP9* gene reference strains exhibited relatively short genetic distance. The results showed that the *NSP9* sequence was highly conserved, consistent with the previous research results.

**Table 1:** Reference sequences of 20 PRRSV NSP9 strains.

**Year**	**Area**	**Strain**	**Genbank**
			**accession number**
1992	USA	VR2332	JX294652
1994	USA	RespPRRS	JX294651
2008	China	CH-1R	EU807840
2011	China	HD	JX162093
2012	China	MM-GD	JX162099
2012	China	YJ-GD	JX162098
2012	China	SH-GD	JX162097
2012	China	GZ-GD	JX162096
2012	China	ZQ-GD	JX162095
2012	China	HuN	JX162094
2012	China	MZ-GD	JX162092
2012	China	QY-GD	JX162091
2012	China	JM-GD	JX162090
2012	China	ZJ-GD	JX162089
2012	China	ZS-GD	JX162088
2012	China	AM-GD-2012	JX826384
2012	China	QW-GD-2012	JX826383
2012	China	JJ-GD-2012	JX826381
2012	China	QQ-GD-2011	JX826380
2012	China	SS-GD-2011	JX826379

Several gene recombination events have been discovered since the NADC30-like strain became the most frequent strain in China; *NSP9* has a recombination hotspot sequence (23). Sun et al. reported regular surveillance and field epidemiological studies for PRRSV across China between 2007 and 2019 (24). Genome recombination of Chinese lineage 1 with two dominant clusters (Chinese HP-PRRSVs: lineage 8.7 and VR2332-like: lineage 5.1) was frequently detected (25). Furthermore, recombination hotspots were discovered near NSP9 and ORF2-4 regions of the PRRSV genome. Comparative analyses of all available genome sequences of North American (NA)-type PRRSV in China and the United States between 2014 and 2018 revealed a high frequency of interlineage recombination hot spots in *NSP9* and the GP2 to GP3 regions (26). Wang et al. indicated that the HNhx strain, isolated from Henan in 2016, was classified into the NADC30-like PRRSV subgroup (27). Recombination analysis revealed that HNhx resulted from the recombination in NSP4 (nt 5261) to NSP9 (nt 7911) between the NADC30 and highly pathogenic PRRSV vaccine strains circulating in China (28). Chen et al. experimentally determined that HBap4-2018 is a new recombinant PRRSV strain, with HP-PRRSV and NADC30-like PRRSV as the main and secondary parent strains, respectively. Five recombination breakpoints were identified in NSP2, NSP3, NSP5, NSP9, and ORF6, respectively, showing a new recombination pattern in the genome (29). Wang et al. compared and analyzed the complete genome sequences of two PRRSV strains, GXYL1403 and GXNN1839 (30). GXYL1403 is a natural recombinant between sublineages 8.1 (CH-1a-like) and 8.3 (JXA1-like). The recombination site of GXYL1403 is located in *NSP9*–*NSP12* (8961–11181 nt). GXNN1839 is a natural recombinant between the lineage 5 (VR-2332-like) and lineage 1 (NADC30-like) strains. The recombination events occurred in *NSP9* (7872-8162 nt) and ORF2 (12587–13282 nt) in the genome of GXNN1839.

## Interaction Between NSP9 and Host Proteins Affects Virus Replication

**Figure 3:**
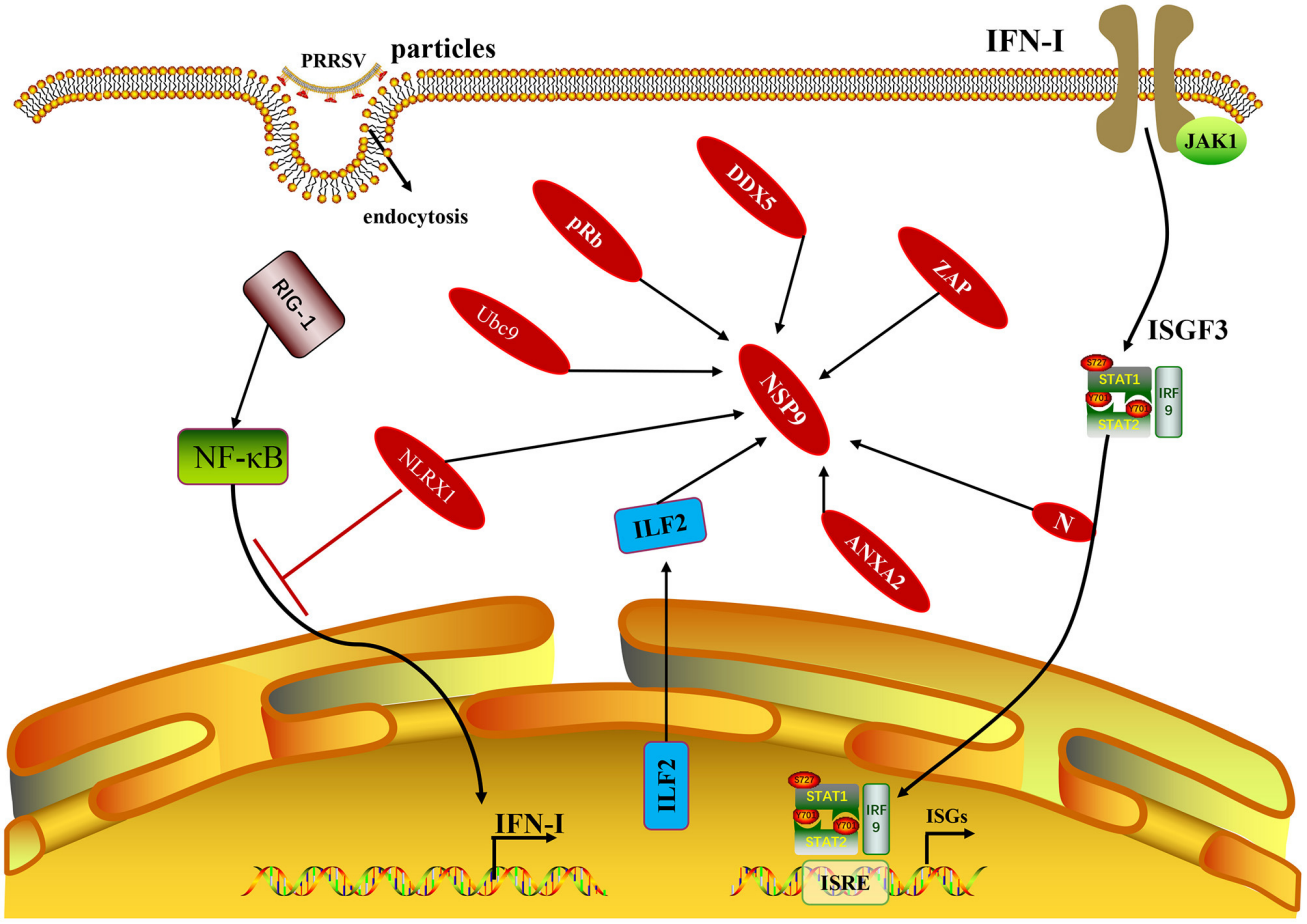
The NSP9 of PRRSV and non-structural proteins with host proteins mentioned in this paragraph. In addition to the host protein, it is also shown that the N protein related to the production of IFN-I also interacts with NSP9.

Some cellular proteins can interact with PRRSV NSP9, and their interactions can regulate viral replication. In MARC-145 cells and porcine alveolar macrophages (PAMs) infected with PRRSV, some cellular proteins, including interleukin-2 enhancer-binding factor 2 (ILF2), can be translocated from the nucleus to the cytoplasm and co-localize with NSP9 and NSP2 of PRRSV (31). In contrast, ILF2 is only located in the nucleus in non-infected cells. ILF2 knockdown is beneficial for the replication of PRRSV, whereas overexpression of ILF2 reduces the replication of PRRSV, indicating that ILF2 plays a negative regulatory role in the replication of PRRSV (32). Only the full-length annexin A2 (ANXA2) can interact with NSP9 after the MARC-145 cells are infected with PRRSV. RNA may contribute to the interaction between NSP9 and ANXA2. The interaction between NSP9 and ANXA2 was reduced by RNase A treatment. In addition, when ANXA2 was knocked out in MARC-145 cells, the growth of PRRSV was significantly blocked, indicating that the interaction between NSP9 and ANXA2 was beneficial to PRRSV replication (33). The zinc-finger antiviral protein (ZAP) is an effective antiviral protein in cells as ZAP inhibits PRRSV infection during the early stages of replication. ZAP interacts with RdRp, and the active sites are in the zinc finger structural domain of ZAP and the N-terminal amino acid 150–160 region of NSP9 (34). Silencing of DEAD-box RNA helicase 5 (*DDX5*) in MARC-145 cells significantly inhibited PRRSV replication. In contrast, overexpression of DDX5 in MARC-145 cells enhanced PRRSV replication, indicating that DDX5 may be a cell cofactor, actively and positively regulating PRRSV replication. DDX5 interacts with NSP9, and the active sites are the DEXDc and HELICc domains in DDX5 and the RdRp domain in NSP9 (35). PRRSV infection promoted the expression of nucleotide-binding oligomerization domain-like receptor (NLRX1), whereas downregulation of NLRX1 expression enhanced PRRSV replication in PAMs. NLRX1 inhibits PRRSV replication by inhibiting RNA synthesis, which is realized by the interaction between the leucine-rich repeat (LRR) domain of NLRX1 and the RdRp domain of PRRSV NSP9 (36). PRRSV NSP9 protein interacts with cell retinoblastoma protein (pRb). NSP9 and pRb are co-localized in the cytoplasm of MARC-145 cells and PAMs infected with PRRSV. Overexpression of the *pRb* gene inhibited PRRSV replication in MARC-145 cells, whereas silencing the pRb gene promoted PRRSV replication in MARC-145 cells. After PRRSV infects MARC-145 cells, NSP9 promotes pRb degradation through the proteasome pathway, demonstrating that the interaction between NSP9 and pRb is beneficial for the replication of PRRSV (37). The NSP1β, NSP4, NSP9, NSP10, and nucleocapsid (N) proteins of PRRSV can interact with the SUMOE2 coupling enzyme Ubc9. Overexpression of Ubc9 inhibited viral genome replication in the early stages of PRRSV infection, whereas siRNA-mediated knockdown of Ubc9 promoted viral replication (38). Understanding the molecular mechanism of the interaction between PRRSV NSP9 and host proteins (Figure 3) can aid in identifying novel antiviral targets and exploring the viral replication mechanism.

## Interaction of NSP9 With Other Viral Proteins Affects the Virus

Nan et al. established a comprehensive schematic diagram of non-structural protein interaction by drawing the interaction network between PRRSV NSPs (39). These interactions are mainly concentrated on NSPs encoded by ORF1b, and the interactions are centered on NSP9, NSP10, and NSP12. These three proteins are mainly interconnected by the transmembrane proteins NSP2, NSP3, and NSP5. NSP9 and NSP12 were identified as two new interaction hubs. Most PRRSV NSPs are related to the viral replication and transcription complex (RTC) in the infection process (40). The membrane-associated NSPs collected by nucleosomes NSP9 and NSP10 in the assembly of the PRRSV RTC may involve the regulatory mechanisms of other viral proteins in the infection process (9). Most of the PRRSV NPSs (NSP1α, NSP1β, NSP3, NSP7α, NSP7β, NSP8, NSP11, and NSP12) can directly bind to NSP9, and the interaction between NSP9 and cytokines may play a role in RNA synthesis in infected cells (41). PRRSV NSP7α interacts with RdRp, and its active site is in the alpha-helix 3 of NSP7α. The mutation of conserved amino acids L69 and F72 causes NSP7α to lose its ability to bind to NSP9, which indicates that these two conserved amino acids are necessary for the interaction between NSP7α and NSP9 (42). The PRRSV N protein binds to NSP9 through protein–protein interactions, and the interaction sites are in two predicted helices formed by 48 residues at the C-terminus of the N protein. Amino acid residues E646, E608, and E611 on NSP9 and amino acid residue E611 on the N protein are predicted to be the critical residues participating in the interaction between N and NSP9. The N protein of PRRSV interacts with the RdRp of NSP9, recruiting the cellular RNA helicase to unravel the double-stranded RNA structure of gRNA, promoting the generation of longer viral sgmRNAs and gRNAs, and participating in the replication and transcription of viral RNA (3).

## NSP9 and Pathogenicity

The HP-PRRSV strain has stronger replication ability and higher pathogenicity than the classical PRRSV strain. However, the molecular mechanism underlying these characteristics is unclear (43). Different HP-PRRSV isolates with high and low pathogenicity were cloned using reverse-genetics technology, and the gene fragments were exchanged between the two strains. The results revealed that NSP9 and NSP10 were the main virulence factors of HP-PRRSV *in vivo*. NSP9 and NSP10, encoded by HP-PRRSV ORF1b, not only affected the replication efficiency of the virus *in vitro* and *in vivo* but were also closely related to the increase in morbidity and the lethal virulence of PRRSV in piglets. This finding helped explain the pathogenesis of HP-PRRSV (5). Two identical amino acid mutations, located at positions 519 and 544 of NSP9, were identified between HP-PRRSV and classical PRRSV strains, respectively. The amino acids at positions 519 and 544 in NSP9 are involved in the replication efficiency of HP-PRRSV and contribute to the enhancement of pathogenicity (20). T586A and T592S of HP-PRRSV NSP9 reduce the replication efficiency of HP-PRRSV and its virulence to piglets, indicating that residues 586 and 592 of NSP9 are the critical sites for determining the lethality of Chinese HP-PRRSV virus (21). These findings significantly contribute to understanding the molecular mechanism of PRRSV replication and pathogenicity and provide a reference to further study PRRSV pathogenicity.

## NSP9 Regulates Cellular Immune Response

NSP9, along with NSP1, NSP2, NSP5, NSP7, NSP10, and NSP11, are involved in the induction of interferon (IFN)-γ in cells and may be involved in cell-mediated immune responses (44). Cao et al. (45) identified many immunostimulatory cytotoxic T lymphocyte (CTL) epitopes by flow cytometry analysis, including two peptides from GP3 and two peptides from NSP9, which significantly improved the production of the degranulation markers CD107a and IFN-γ in cytotoxic CD4+ T cells, CD8+ T cells, and γδ T cells. NSP9-TMP9 was identified as an immunogenic epitope, which can stimulate the proliferation of specific CD8+ T cells and CTLs, and the expression of IFN-γ in SPF Landrace pigs carrying the SLA-1^*^1502 allele (46). Autophagy is an important mechanism to maintain cell homeostasis, associated with stress responses, such as immune response and inflammation. It is also an innate defense mechanism against infectious pathogens (47). PRRSV infection can induce autophagy, which can promote PRRSV replication. The PRRSV NSP3, NSP5, and NSP9 can activate intracellular autophagy, and NSP3 and NSP5 induce the formation of autophagic bodies, primarily the cytoplasmic domain of NSP3 (42). However, the specific role of NSP9 in autophagy needs further experimental verification.

## Compound Drugs and Modifications Against NSP9 Affect Virus Replication

The A283 and H421 residues of PRRSV NSP9 play a key role in PRRSV replication by affecting the fidelity of its RdRp and are closely related to the sensitivity of PRRSV to ribavirin (48). The actual usage frequency of each codon pair is greater than the statistical prediction, and such codon pair bias (CPB) affects gene translation. Gao et al. “recombined” the existing codons of HP-PRRSV genes *GP5, M, NSP2*, and *NSP9*, which made the CPB of these genes more negative (49) the results showed that the optimization of RdRp significantly reduced PRRSV replication in PAMs. PRRSV infection depends on the existence of lipid rafts in the cell membrane and the virus envelope. Cell lipid rafts play a vital role in the entry, replication, and release of PRRSV. The structure of virus lipid rafts is essential to maintain the integrity of the virus particle core to ensure the infectivity of PRRSV. NSP9 is distributed in the raft, indicating that the raft may be a platform for PRRSV replication; thus, small molecules disturbing the membrane lipid raft can also provide a means for developing antiviral drugs (50). Xie et al. constructed an siRNA expression vector targeting the NSP9 region of PRRSV. This study showed that NSP9, an essential polymerase protein in PRRSV, can be used as an effective antiviral targeting gene (51). Furthermore, siRNA expression vectors, pSi-294 and pSi-1488, can effectively inhibit the expression of the NSP9 protein of PRRSV and consequently inhibit PRRSV replication. Therefore, siRNAs can be valuable in developing new antiviral strategies and offer a candidate drug for basic research on PRRSV. DNA-based short antisense oligonucleotides (AS-ON) can effectively target PRRSV NSP9, thus inhibiting PRRSV replication in MARC-145 cells and PAMs (52). Two types of AS-ONs, modified by locked nucleic acids (LNAs), are more effective in reducing the cytopathic effect (CPE) induced by PRRSV and maintaining cell viability. In a cell culture model, LNA modification prolonged the lifespan of AS-ON. Incorporating LNA into AS-ON technology has a higher therapeutic prospect for PRRS control (53). The water extract from the fruiting body of *Cryptococcus obliquus* significantly inhibited PRRSV infection by inhibiting PRRSV entry, RNA expression, protein synthesis, cell-to-cell transmission, and PRRSV particle release. However, it did not block the combination of PRRSV and cells. The water extract from the fruiting body of *Cryptosporus* could directly inhibit the activity of PRRSV RdRp, thus interfering with the synthesis of PRRSV RNA and proteins. The extract effectively inhibited HP-PRRSV infection *in vivo*, reduced the viral load in serum, and improved the survival rate of pigs infected with HP-PRRSV, indicating that *Cryptosporus* water extract has potential as an anti-PRRSV agent (54). Camel single-domain antibodies (nanoparticles) represent a promising antiviral method because of their small size, high specificity, and good solubility. The PRRSV NSP9 is crucial for virus replication, and its sequence is relatively conserved, making it a logical antiviral target of PRRSV. NSP9-specific nanoparticles (Nb6) were isolated from a phage display library (VHH) containing only the variable region of the chain antibody of Camellidae heavy. Nb6 maintained its antigen-binding ability when expressed in the cytoplasm. Intracellular Nb6 effectively inhibited PRRSV replication by inhibiting PRRSV genome replication and transcription. Nb6 could also protect MARC-145 cells from a virus-induced CPE and completely block PRRSV replication at a multiplicity of infection of 0.01 or lower (55). Enzyme-linked immunosorbent assay (ELISA) showed that residues Ile588, Asp590, Leu643, Tyr62, Trp105, and Pro107 of Nb6 were the critical sites in the interaction between NSP9 and NB6. Structural analysis of the NSP9–Nb6 complex revealed new amino acid interactions involved in viral RNA replication, which will help guide the development of structure-based anti-PRRSV drugs (4). The cells expressed in prokaryotic expression systems could penetrate MARC-145 cells and PAMs in a time-and dose-dependent manner and inhibited the replication of PRRSV genotype 1 and 2 strains. In addition, Nb6 binds to the C-terminal region of NSP9, which contains two discontinuous regions, NSP9454-551 and NSP9599-646, and overlaps with the predicted catalytic domain of RdRp. These data indicate that TAT-Nb6 has high development potential as an anti-PRRSV drug (41).

## Application of NSP9 in Vaccines

NSP9 encodes RdRp in PRRSV, and RdRp only appears in the virus replication process and does not exist in host cells. Therefore, antigens targeting the RdRp fusion protein can distinguish wild strains from inactivated vaccines (18). Immunostimulatory type I CTL epitopes of PRRSV are essential for vaccine development. Many immunostimulatory CTL epitopes have been identified, including two peptides from GP3 and NSP9 and two that inhibit IFN-γ production. These CTL epitopes are expected to contribute to future vaccine development against PRRSV (45, 56). The epitope peptide NSP9-TMP9, based on NSP9, has been identified as a CTL epitope of PRRSV with strong immunogenicity. NSP9-TMP9 can enhance the immune response and produce a specific CTL response. This indicates that the peptide vaccine can confer effective immune protection, demonstrating its immense potential in the development of an effective epitope vaccine (46). Paride et al. identified two highly conserved heptapeptide T cell epitopes in NSP9, which provided an important scientific reference for developing immune agents that can provide extensive cross-protection against various PRRSV strains (57).

## Application of NSP9 in Detection Methods

Spear et al. established a copy-specific reverse transcription-quantitative polymerase chain reaction (RT-qPCR) detection method based on PRRSV-2 isolates. This technology is aimed at the RNA-dependent RNA polymerase gene (NSP9), which is unique to gRNA and does not exist in subgenomic and heteronuclear RNA (58). This method has proven to be linear in nine orders of magnitude (10^10^-10^2^ copies) and can be easily applied to detecting multiple PRRSV strains. This method will significantly improve the ability to evaluate and compare PRRSV replication in various tissues and among different strains, including highly pathogenic strains that are of great concern to the global pork industry. Kang et al. established a closed ELISA (b-ELISA) to detect PRRSV NSP9 with the monoclonal antibody 2D6. Since b-ELISA only detects antibodies produced by active PRRSV replication, such as natural infection or live vaccine inoculation, this method can distinguish between antibodies against live and inactivated vaccines in pigs (59).

## Summary

NSP9 of PRRSV is the core component of the RTC. As the critical enzyme in viral RNA synthesis, its RdRp function is crucial for PRRSV replication. It is the key factor regulating nucleotide selectivity and fidelity in the viral replication process. NSP9 participates in various immunosuppressive reactions. As a newly discovered protein interaction hub, NSP9 interacts with the host cell and self-proteins to regulate virus replication, which is closely related to the improvement of replication efficiency and the formation of pathogenicity of HP-PRRSV. NSP9 can be used as an effective antiviral targeting gene to develop new antiviral strategies and lay a foundation for developing new vaccines and antiviral drugs. The NSP9 gene sequence is highly conserved, and amino acid similarity among different PRRSV strains is high. Based on these characteristics, new and efficient detection reagents, as well as methods for PRRSV, can be developed in the future.

However, PRRSV is constantly undergoing mutation and recombination, which poses significant challenges to vaccine research and development. Currently, the effect of attenuated and inactivated vaccines against PRRSV is not optimal. Therefore, developing new therapeutic strategies with effective antiviral activity is urgently required. PRRSV infections are also characterized by persistent infection, immunosuppression, and complex immune mechanisms. Co-infection with other pathogens increases the mortality rate of pigs. The infection also slows down the growth of pigs, increases feeding costs, reduces production performance, and brings heavy economic losses to the pig industry. Research on the molecular biology of PRRSV is currently progressing rapidly. This research focuses on the screening, transformation, and utilization of immunogenic and pathogenic genes. Understanding the structure of each PRRSV genome and the process of replication in cells is the basis and key to its prevention and control. PRRSV NSP9 plays a vital role in viral replication and virulence. Studying the molecular pathogenic mechanism of PRRSV NSP9 will provide a scientific basis for preventing and controlling this disease. The comprehensive overview of PRRSV NSP9 provided in this review will help further explore the relationship between the virus and its host and offers a reference for the prevention and control of PRRS and the development of new vaccines and antiviral drugs.

## Author Contributions

HS and HZ collect data and write the original draft. YC and LH complement and improve the manuscript. MZ and NW made the final revision of the manuscript. All authors read and approved the final manuscript.

## Funding

This study was supported by the National Natural Science Foundation of China (31902279), Guang Dong Basic and Applied Basic Research Foundation (Nos. 2019a15110056 and 2021A1515110322), and Guangdong Science and Technology Program Project (2017A020208079 and 2021A1515012388).

## Conflict of Interest

The authors declare that the research was conducted in the absence of any commercial or financial relationships that could be construed as a potential conflict of interest.

## Publisher's Note

All claims expressed in this article are solely those of the authors and do not necessarily represent those of their affiliated organizations, or those of the publisher, the editors and the reviewers. Any product that may be evaluated in this article, or claim that may be made by its manufacturer, is not guaranteed or endorsed by the publisher.
